# PERSPECTIVES OF CAREGIVERS TO BLACK AND HISPANIC PERSONS LIVING WITH DEMENTIA (PLWD) ABOUT END-OF-LIFE COMMUNICATION

**DOI:** 10.1093/geroni/igad104.0341

**Published:** 2023-12-21

**Authors:** Zainab Toteh Osakwe, Sasha Perez, Michelle Odlum, Ana Stefancic

**Affiliations:** Adelphi University, Garden City, New York, United States; Icahn School of Medicine at Mount Sinai, New York City, New York, United States; The George Washington University, Washington, District of Columbia, United States; Columbia University, New York City, New York, United States

## Abstract

Black and Hispanic Persons Living with Dementia (PLWD) heavily rely on home healthcare to remain comfortably in their own home, even at the end of life. Despite growth in use of hospice in the U.S., research demonstrates persistent racial differences in utilization of hospice - a key indicator of high-quality end of life care. This is particularly relevant among older Black and Hispanic PLWD who experience additional burdensome interventions at the end of life which often run counter to their goals of care. This study examined perceptions of caregivers to Black and Hispanic PLWD regarding discussions with home healthcare nurses about end-of-life communication and transitions to hospice. We conducted semi-structured interviews with 19 Black and Hispanic family caregivers of PLWD. Interviews were recorded, transcribed, translated and analyzed using conventional content analysis. Results showed that Black and Hispanic caregivers of PLWD do not prioritize aggressive and burdensome care at the end of life, had low expectations of the healthcare system to support their needs and preferences at the end-of-life, and were willing to discuss and accept hospice if discussions were transparent about resources and entitlements; acknowledged the strengths of the PLWD/caregivers; involved positive and respectful communication with families during a home visit; acknowledged respect for their social communities and home environment; sat down during a home visit and recognized the role of religion at the end-of-life. Implications to improve care include incorporating family caregiver discussions, identify social determinants of health, and address the role of religion in future interventions.

